# Tacrolimus (FK-506) levels determined by 2 different chemiluminescence immunoassay-based analytical methods: A comparative cross-sectional study

**DOI:** 10.1097/MD.0000000000044895

**Published:** 2025-09-26

**Authors:** Vijay Kumar Sharma, Apeksha Niraula, Eans Tara Tuladhar, Raju Kumar Dubey, Aseem Bhattarai, Mithileshwer Raut, Anuradha Kadel, Nikita Kharal, Srijana Sapkota, Prakash Pokhrel

**Affiliations:** a Department of Clinical Biochemistry, Institute of Medicine, Maharajgunj Medical Campus, Kathmandu, Nepal.

**Keywords:** chemiluminescence immunoassay, immunoassays, kidney transplantation, tacrolimus, therapeutic drug monitoring

## Abstract

Several assays have been used to monitor tacrolimus levels. Automated immunoassay systems are preferred routinely because of their simplicity and rapid turnaround times. However, variations in the measured results for the same sample by different assays necessitate the evaluation of various immunoassay systems for consistency. This study aimed to compare whole-blood tacrolimus concentrations measured using 2 different chemiluminescence analytical methods: chemiluminescent microparticle immunoassay (CMIA; Abbott Diagnostics) and chemiluminescent immunoassay (CLIA; Snibe Diagnostics). A total of 257 patients who had undergone kidney transplantation were included in this study. Whole-blood tacrolimus concentrations were measured using the MAGLUMI 800 analyzer and compared with the ARCHITECT i1000SR analyzer using correlation analysis and Bland-Altman plots. There was a strong correlation between tacrolimus concentrations measured using MAGLUMI and those measured using ARCHITECT (*R* = 0.916, *P* < .001). An intraclass correlation coefficient (ICC) value of 0.934 (CI = 0.915–0.949) indicated excellent reliability of the measurements. Comparison of samples with a routine ARCHITECT assay for tacrolimus showed a minimal bias of 0.385 ng/mL (95% CI = 4.46%–3.69%), but scattering analysis indicated that the method agreed when the tacrolimus levels were low but disagreed at high levels. Overall, the measured tacrolimus level in patient samples was higher by MAGLUMI than by ARCHITECT with a median difference of 0.17 ng/mL and a median percentage difference of 3.4% respectively. Snibe MAGLUMI is a reliable assay with excellent correlation, but a small positive bias against Abbott ARCHITECT in measuring whole-blood tacrolimus levels. This bias is most likely due to the cross-reactivity of the metabolites in the Snibe MAGLUMI platform.

## 1. Introduction

Kidney transplantation is the treatment of choice for end-stage renal diseases (GFR < 15 mL/min/1.73 m^2^).^[1]^ Patients who undergo kidney transplantation require lifelong immunosuppression. The maintenance of immunosuppression includes a combination of medications, with tacrolimus-based regimens being the top choice.^[2]^

Tacrolimus, or FK-506 is an immunosuppressant macrolide discovered by Fujisawa Pharmaceutical Co. Ltd (Tsukuba, Japan) in 1984.^[3]^ In vivo, tacrolimus binds to a family of proteins called FKBP (FK-binding proteins), forming a pentameric complex with Ca^2+^, calmodulin, and calcineurin. The resulting complex inhibits the action of the nuclear factor of activated T-cells (NFAT), the expression of which is required for interleukin-2 (IL-2) to initiate signaling the activation of T-lymphocytes.^[4]^ Compared with cyclosporine, Tacrolimus produces 50 to 100 times higher in vitro inhibitory action against T-cells and is useful for the prophylaxis of graft-versus-host disease when used alone or in conjunction with other immunosuppressive drugs.^[5,6]^

Therapeutic drug monitoring (TDM) of tacrolimus is an integral part of optimizing drug therapy in transplant patients to individualize drug doses because of its narrow therapeutic index and high inter-patient variability in pharmacokinetics.^[7]^ Implementation of the wrong drug dose may cause graft rejection if underdosed, or when overdosed, nephrotoxicity, neurotoxicity, and many other adverse effects.^[8]^ In 2019, the Immunosuppressive Drugs Scientific Committee of the International Association of Therapeutic Drug Monitoring and Clinical Toxicity (IATDMCT) released an updated consensus report on TDM for tacrolimus, covering advancements in pharmacokinetics, pharmacogenetics, pharmacodynamics, and immunologic biomarkers, to assist health care professionals in handling this immunosuppressive drug in solid organ transplantation.^[9]^ Since immunosuppressive drugs are used on a long-term basis, whole blood or plasma concentration measurement of immunosuppressive drugs in organ transplant patients will help to ensure compliance with the drug treatment protocol.^[10]^ In the clinical setting, immunoassays are used to measure the tacrolimus concentration in whole blood.

In current clinical practice, a number of assay methods are used to monitor tacrolimus levels, including enzyme linked immunosorbent assay (ELISA), microparticle enzyme immunoassay (MEIA), chemiluminescent microparticle immunoassay (CMIA), high-performance liquid chromatography (HPLC), liquid chromatography-tandem mass spectroscopy (LC/MS/MS) and homogeneous enzyme multiplied immunoassay technique (EMIT).^[6]^ Accurate and precise assays for the measurement of whole-blood tacrolimus levels need to be employed. Methods based on HPLC and HPLC-MS have become the gold standard for this purpose because of their proven accuracy; however, complexity and initial costs have led to the frequent adoption of automated immunoassays.^[11]^ Automated immunoassay systems that can measure tacrolimus levels in a short time are used for routine monitoring of blood tacrolimus concentrations. However, it has been reported that the results for the same sample vary when measured using different assay systems.^[12]^ Therefore, there is an increasing need to evaluate different immunoassay systems for consistency. The reagents used in automated immunoassay systems have been improved over the years. Therefore, there might be an improvement in the specificity, extraction efficiency, and stability of blood concentrations of tacrolimus measured by the current assay systems from those reported previously.^[13]^ CMIA (Abbott Laboratories, Abbott Diagnostics, Chicago) is the most commonly used method for monitoring tacrolimus in Nepal, which allows rapid determination of tacrolimus in whole blood.

MAGLUMI Tacrolimus is a recently developed assay from Snibe Diagnostic (Snibe Co., Shenzhen, China), which is based on a chemiluminescence immunoassay (CLIA). MAGLUMI FK-506 is an in vitro chemiluminescence immunoassay for the quantitative determination of FK-506 in human whole blood, which has a simple method of sample preparation, reasonably low cost of conducting tests, and the inclusion of controls and calibrators with the reagents, thereby obviating the need for their separate purchase. In the case of developing countries such as Nepal, the advantages of MAGLUMI FK-506 might contribute to lessening the financial burden on patients. Therefore, the present study aimed to determine the correlation and compare the agreement of results from 2 different chemiluminescence analytical methods that are commercially available in Nepal, namely chemiluminescent microparticle immunoassay (CMIA; Abbott Diagnostics) and Chemiluminescent Immunoassay (CLIA; Snibe Diagnostic) in measuring tacrolimus level.

## 2. Methods

### 2.1. Study design and setting

This was an analytical cross-sectional study conducted from November 1, 2023, to May 31, 2024, in the Department of Clinical Biochemistry of Tribhuvan University Teaching Hospital (TUTH). A total of 257 whole-blood samples from kidney transplant patients receiving tacrolimus treatment at Tribhuvan University Teaching Hospital (TUTH) (Kathmandu, Nepal) were enrolled in the study. Ethical approval was obtained from the Institutional Review Committee of the Institute of Medicine, Maharajgunj, Kathmandu (Ref. No.: 227(6-11) E2 080/081).

### 2.2. Tacrolimus assays

#### 2.2.1. ARCHITECT i1000SR analyzer

Tacrolimus levels in whole blood are tested routinely in TUTH by an ARCHITECT i1000SR analyzer (Abbott Diagnostics). This method employs chemiluminescent microparticle immunoassay (CMIA), in which the pretreated sample, assay diluent, and anti-tacrolimus-coated paramagnetic microparticles are combined to create a reaction mixture. The tacrolimus present in the sample binds to the anti-tacrolimus-coated microparticles. After a delay, tacrolimus acridinium-labeled conjugate is added to the reaction mixture. The tacrolimus on the acridinium-labeled conjugate competes for the available binding sites on the microparticles. Finally, the resulting chemiluminescent reaction is measured as relative light units (RLUs) after the addition of Pre-Trigger and Trigger Solutions. There is an indirect relationship between the amount of tacrolimus in the sample and RLUs detected by the ARCHITECT iSystem Optics.

The analytical measurement range for Abbott ARCHITECT tacrolimus immunoassay is 2 to 30 ng/mL. The minimum reportable value of 2 ng/L is based on the claimed functional sensitivity of the Abbott ARCHITECT method. The limit of detection is ≤ 1.5ng/mL. Six calibrators of different levels were available: 0, 3, 6, 12, 20 and 30 ng/L. Tri-level controls (6.63ng/mL,11.0ng/ mL, 24.8ng/mL) were analyzed with each run to confirm assay precision. The between-day precision was defined as the coefficient of variation. As all pretreated specimens should be tested within 30 minutes, the assay was performed each day in batches depending on the number of specimens received.

#### 2.2.2. MAGLUMI 800 analyzer

The 257 whole-blood samples were re-tested using the MAGLUMI 800 analyzer (Snibe Diagnostics) immediately after being analyzed by the ARCHITECT i1000SR analyzer. Maglumi FK-506 assay employs a competitive chemiluminescence immunoassay. The first step includes the extraction of red blood cell lysate from whole blood by the use of a pretreatment reagent. The pretreated sample, buffer, and magnetic microbeads coated with anti-FK-506 monoclonal antibody are mixed thoroughly and incubated. Then, ABEI labeled with purified FK-506 antigen is added, forming antigen-antibody complexes. The supernatant is decanted after precipitation in a magnetic field and a wash cycle is performed. Subsequently, the Starter 1 + 2 is added to initiate a chemiluminescence reaction. The light signal is measured by a photomultiplier as RLUs, which is inversely proportional to the concentration of FK-506 in the sample.

The analytical measurement range for the Snibe MAGLUMI FK-506 immunoassay is 0.1 ng/mL to 100 ng/mL. Two-point calibration was performed, and single internal quality control (20.0 ng/mL) was analyzed with each run to confirm assay precision. Between-day precision was defined as the coefficient of variation. Between-run precision for the FK-506 quality control samples based on guidance from CLSI EP5-A2 ranged from 2.17% for the control 1 (mean concentration 20.324 ng/mL) to 0.74% for the control 2 (mean concentration 50.150 ng/mL). Both assays require several pre-analytical steps to guarantee the appropriate lysis of the erythrocytes for accurate Tacrolimus measurement.

### 2.3. Statistical analysis

All data were analyzed using Excel (Microsoft) and SPSS statistical package version 23 (SPSS Inc, Chicago). Continuous variables were described as means with standard deviations or as medians with an interquartile range depending on their distribution. Spearman correlation was used to measure the correlation between the 2 systems. The Bland-Altman plot was used to analyze the agreement between the 2 systems. Analysis of the bias and variability of differences in tacrolimus values between the 2 methods was performed using the Bland-Altman method. The difference between the measurements obtained from the 2 methods was calculated and plotted against the mean of the 2 measurements.

To assess the reliability and reproducibility of the measurements, we employed the intraclass correlation coefficient (ICC). The ICC estimates and their corresponding 95% confidence intervals (CI) were calculated using SPSS statistical package version 23 (SPSS Inc). The ICC was calculated based on the mean rating, absolute agreement, and 2-way mixed-effects model. The reliability of the measurement was derived based on ICC values as: poor reliability (<0.5), moderate reliability (0.5–0.75), good reliability (0.75–0.9), and excellent reliability (>0.9).^[14]^ *P*-value of < 0.05 was considered to be statistically significant.

## 3. Results

**Figure 1.**
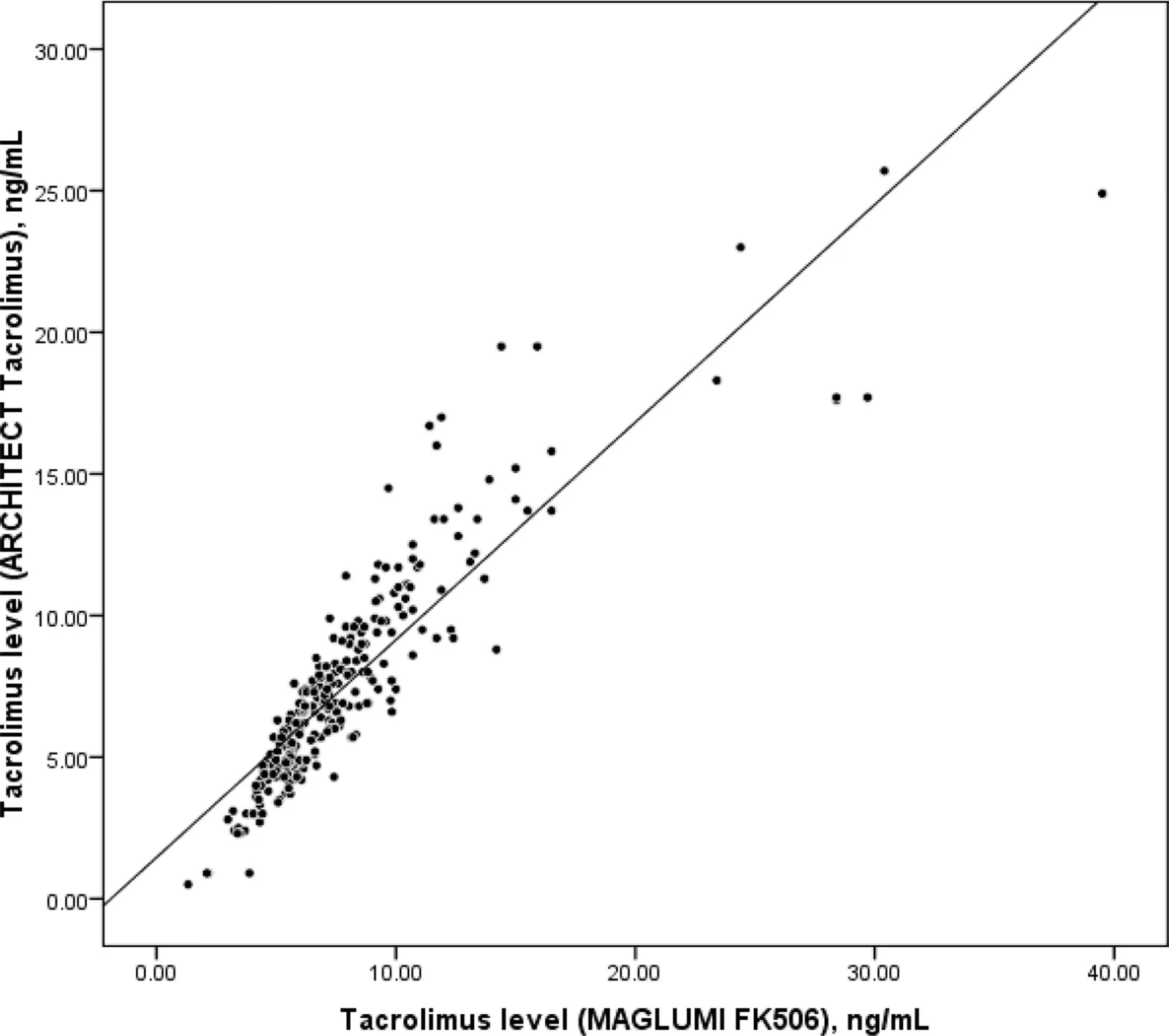
Scatter plots and regression line correlating blood tacrolimus concentrations (ng/mL) obtained by ARCHITECT with those obtained by MAGLUMI.

A total of 257 samples received for the tacrolimus assay were analyzed across the 2 immunoassay systems. Of the 257 stable renal transplant recipients recruited, 197 (76.7%) were male and 60 (23.3%) were female. The mean age was 38.06 ± 10.74 years. The median (IQR) tacrolimus levels in MAGLUMI and ARCHITECT are shown in Table 1. Spearman rho correlation between the 2 assay methods showed a strong correlation as depicted in Table 1 and Figure 1.

**Table 1:** Spearman correlation for tacrolimus level measured by MAGLUMI 800 analyzer and ARCHITECT i1000SR.

Parameter	MAGLUMI FK-506	ARCHITECT tacrolimus	Correlation coefficient	*P*-value
Tacrolimus	6.87 (5.5,9.07)	6.80 (4.9,9.2)	0.916	<.001**

* Correlation is significant at they 0.01 level (2-tailed).

To assess the agreement between MAGLUMI and ARCHITECT results, the difference between the 2 methods was calculated (Mean of MAGLUMI FK-506 – Mean of ARCHITECT Tacrolimus result). A mean difference of 0.385, with a standard deviation of 2.08 was obtained as shown in Table 2. To measure the reliability that reflects both the degree of correlation and agreement between measurements, the ICC was used. ICC value of tacrolimus was 0.934 (*P* < .001) which indicates excellent reliability. ICC estimates and their 95% confidence interval (CI) for tacrolimus across the 2 assays are shown in Table 2.

**Table 2:** Mean difference and intraclass correlation coefficients between MAGLUMI FK-506 and ARCHITECT tacrolimus.

Parameter	Mean of MAGLUMI FK-506	Mean of ARCHITECT tacrolimus	Mean difference (MAGLUMI FK-506-ARCHITECT tacrolimus)	ICC (confidence interval)	*P*-value
Tacrolimus	7.97	7.58	0.385	0.934 (0.915–0.949)	<0.001**

* Correlation is significant at the 0.01 level (2-tailed).

**Figure 2.**
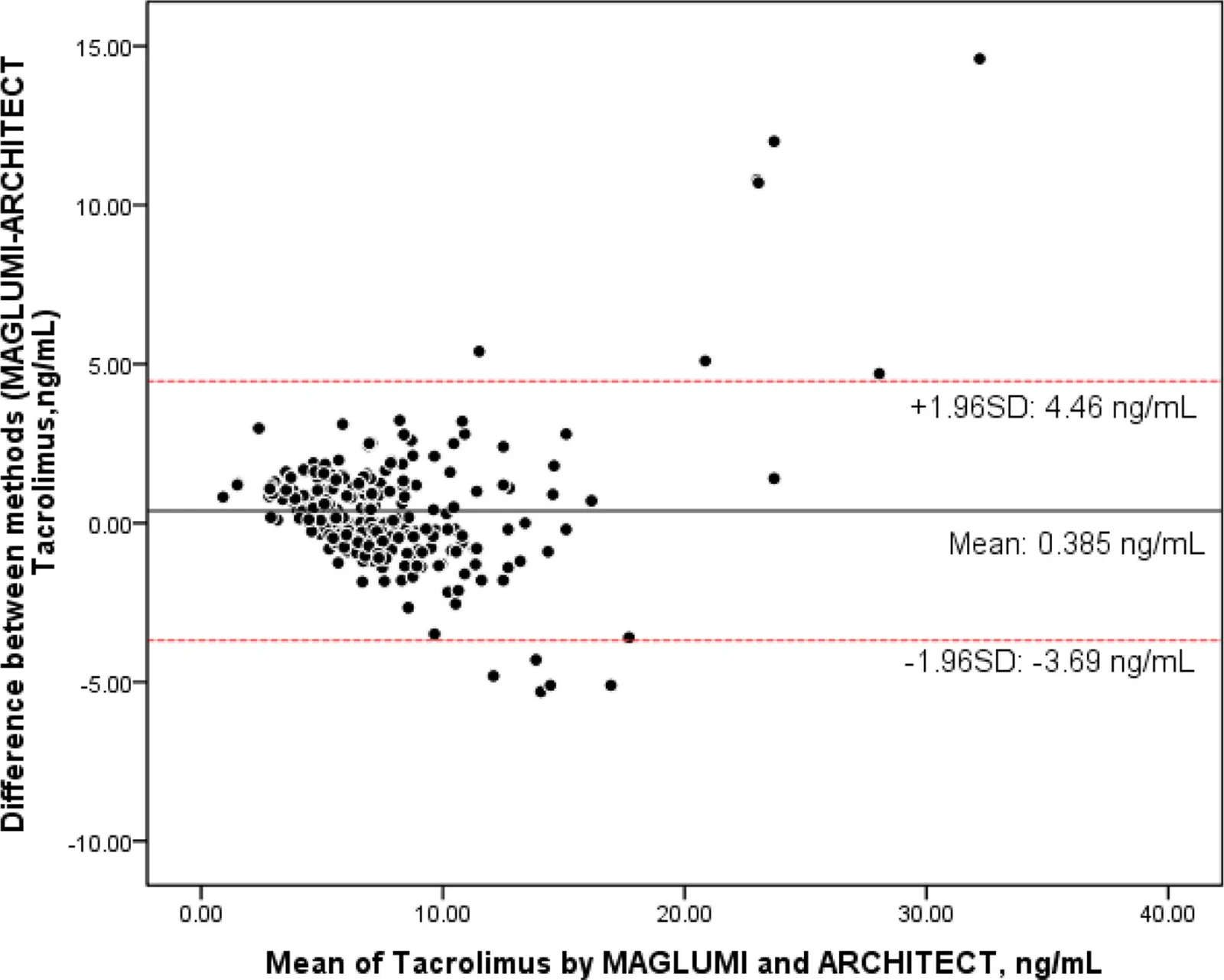
Bland-Altman plots of MAGLUMI FK-506 against ARCHITECT Tacrolimus.

Blood tacrolimus concentrations measured by MAGLUMI were found to be significantly higher than those measured by ARCHITECT, with a median difference of 0.17 ng/mL (*P* < .01). The percentage difference (%diff) was calculated as the difference between the 2 methods as a percentage of the ARCHITECT value. The median %diff was 3.4%. A bias analysis of the MAGLUMI FK-506 versus ARCHITECT Tacrolimus assay was performed and scatter plots were generated by plotting the differences between the 2 assays. The values were then plotted against the mean of the 2 measurements using the Bland-Altman method. The average ng/mL difference bias exhibited by MAGLUMI FK-506 versus ARCHITECT Tacrolimus assay in this study was 0.385 ng/mL which is close to zero and hence showed a very small bias. Scattering analysis indicated that the method agreed when tacrolimus levels were low but disagreed at high levels. The 95% limits of agreement for tacrolimus values in the given samples were determined to be from 4.46 to −3.69, as shown in Figure 2.

## 4. Discussion

This was the first study to measure the analytical performance of the novel MAGLUMI FK-506 and ARCHITECT tacrolimus under routine conditions in Nepalese renal transplant recipients. In this study, we compared 2 methods for measuring tacrolimus concentrations in whole blood. The present study revealed a strong correlation between the 2 methods. The reliability of the measurements was evaluated using the ICC, where tacrolimus exhibited ICC values above 0.90, indicating excellent reliability. Agreement with the Architect platform was close to zero, showing a small bias in lower values of tacrolimus; however samples in the higher concentration ranges were more dispersed with a low level of agreement, but there were too few of these samples to draw any conclusions.

HPLC-MS/MS is a reference method for measuring whole-blood tacrolimus concentration, but its use has been limited by high instrumental cost, the need for expertise, and limited laboratory infrastructure in small-scale laboratories, especially in developing countries such as Nepal. Therefore, user-friendly automated immunoassays have been adopted as simple and convenient alternatives for measuring tacrolimus concentrations.^[15,16]^ Despite having the advantage of a short turnaround time, the measured results of different immunoassay systems for the same sample vary.^[17]^ Therefore, if a patient moves to another hospital, the blood tacrolimus concentrations cannot be compared if both hospitals use different immunoassays. This increases the need to evaluate the consistency and homogeneity of different immunoassay systems.

The CMIA (Abbott Laboratories) method has been widely used in Nepal^[18]^ for monitoring whole-blood tacrolimus concentration in transplanted patients. Previous studies have reported a comparison of Tacrolimus concentrations in transplant patients between CMIA and other methods.^[19–21]^ In this study, we compared tacrolimus levels measured using MAGLUMI FK-506 with routinely established ARCHITECT tacrolimus and observed a high correlation in whole-blood concentrations, as indicated by a correlation coefficient of 0.916. In addition, this study demonstrated excellent reliability.

In this study, we also confirmed the overestimation of whole-blood tacrolimus concentration by MAGLUMI compared to that measured by ARCHITECT. The median difference between the 2 assays was 0.17 ng/mL, corresponding to a median overestimation of 3.4 % by MAGLUMI. The difference in immunoassay results and overestimation of tacrolimus in patient samples analyzed by the MAGLUMI assay most likely represents the contribution of cross-reacting anti-tacrolimus monoclonal antibodies with active and inactive drug metabolites of tacrolimus present in the patient’s whole blood, in particular 31-O-dimethyl (M-II), 15-O-demethyl (M-III), and 15,31-O-dimethyl (M-V).^[16,22]^ A study by AK Gonschior and his colleagues in Germany pointed out that tacrolimus metabolites could add up to 44.8% of the tacrolimus blood concentration (range 16%–152%) in kidney transplant recipients which can cause an unpredictable overestimation of the true drug concentrations.^[23]^ Tacrolimus is metabolized by the cytochrome P450 isoenzymes 3A4 and 3A5 (CYP3A4/5) system and several drugs and other variables such as pharmacogenetic variability in CYP3A4/5, age, disease conditions, and time since transplant affect the expression of this enzyme. Metabolites can cross-react with antibodies in immunoassay platforms, causing specificity issues that lead to overestimation of tacrolimus levels.^[24]^ The reported overestimation of tacrolimus concentrations by immunoassays in renal transplant recipients in other studies has ranged from 18% to 48%.^[25,26]^

In addition, an Italian study has explained that the possible reason for a higher value of tacrolimus determined by immunoassay during the early-post surgery period in patients can be the low plasma concentrations of albumin.^[27]^ The correlation of albumin with the inter-assay differences may possibly be explained by the presence of unbound tacrolimus or metabolites with a lower affinity for albumin that are still able to cross-react with the antibody.^[28]^

Good agreement between the 2 methods was observed at lower values with minimal bias. Samples in the higher concentration ranges were more dispersed, but there were too few samples to draw any conclusions. The mean bias was 0.385 ng/mL with the 95% limits of agreement for tacrolimus values from 4.46 to −3.69. The positive bias of 0.385 ng/mL in Maglumi as compared to ARCHITECT could be due to the differential calibration of both Snibe Maglumi and Abbott ARCHITECT and between-method bias that may change with time, as observed for the sirolimus microparticle enzyme immunoassay.^[29]^ Lastly, the comparison of analytical procedures is hampered by the lack of standard reference methods and certified reference materials.^[30]^ Tacrolimus is the only immunosuppressive drug for which a whole blood certified reference material (ERM-DA110a) is available. Therefore, as advised by the IATDMCT committee, clinicians should be aware of the potential pitfalls when interpreting results, and continuous education is recommended.^[31]^ A study from a health center in the United States determined a mean bias of 0.36 µg/L (ng/mL) in immunoassay like Abbott ARCHITECT i2000 compared to LC-MS/MS in the analytical measurement range of 2 to 30 µg/L.^[32]^ Similarly, a multi-platform comparison of tacrolimus quantification by 2 immunoassays and 3 LC-MS/MS assays in a total of 216 specimens showed a positive absolute bias of 2.1 by Abbott ARCHITECT.^[33]^ The reason for this significant inter-assay variability between immunoassays and LC-MS/MS, and even within different immunoassay methods may be associated with the fact that immunoassay measures the parent drug and its metabolites, while LC-MS/MS measures only the parent drug.^[33]^ However, there is no available data regarding the biases of Snibe Maglumi.

This single-centered retrospective study limits the applicability of our results to a broader transplant population. Because LC-MS/MS is the reference assay for TDM and is commonly employed to rule out potential interference effects from drug metabolites, a comparison with LC-MS/MS would have been advantageous. However, immunoassays are widely used in Nepal, particularly among stable transplant outpatients. To the best of our knowledge, this is the first study of its type to compare 2 different but highly prevalent approaches for monitoring tacrolimus levels in Nepal.

## 5. Conclusion

In conclusion, Snibe Maglumi is a reliable assay with excellent correlation, but a small positive bias against Abbott ARCHITECT. This bias is most likely due to a combination of cross-reactivity of metabolites in the Snibe Maglumi platform and lack of standardization. The improvement in turnaround time for locally available assays may improve the time taken for clinical decisions and enhance patient outcomes. However, clinicians should be mindful of the potential for discordant clinical decisions between the 2 methods at the limits of the target range, particularly in newly transplanted patients.

## Acknowledgments

The authors acknowledge all the laboratory staff of the Biochemistry Laboratory, Tribhuvan University Teaching Hospital for their genuine support. The reagents used to conduct tacrolimus immunoassays were provided by Snibe Diagnostics (Shenzhen, China). The manufacturer has no relationships and has not influenced the work or anything related.

## Author contributions

**Conceptualization:** Vijay Kumar Sharma, Apeksha Niraula, Eans Tara Tuladhar.

**Data curation:** Vijay Kumar Sharma, Anuradha Kadel.

**Formal analysis:** Vijay Kumar Sharma, Raju Kumar Dubey, Mithileshwer Raut.

**Investigation:** Anuradha Kadel, Nikita Kharal, Srijana Sapkota, Prakash Pokhrel.

**Methodology:** Vijay Kumar Sharma, Raju Kumar Dubey, Aseem Bhattarai, Mithileshwer Raut.

**Software:** Vijay Kumar Sharma, Apeksha Niraula, Aseem Bhattarai.

**Writing – original draft:** Vijay Kumar Sharma, Anuradha Kadel.

**Writing – review & editing:** Vijay Kumar Sharma, Apeksha Niraula, Eans Tara Tuladhar.
